# Calcified thrombi of the Valsalva sinuses mimicking an aortic valve tumour

**DOI:** 10.1093/icvts/ivac232

**Published:** 2022-09-02

**Authors:** Ryo Nakamura, Kentaro Honda, Hideki Kunimoto, Yoshiharu Nishimura

**Affiliations:** Department of Thoracic and Cardiovascular Surgery, Wakayama Medical University Hospital, Wakayama City, Japan; Department of Thoracic and Cardiovascular Surgery, Wakayama Medical University Hospital, Wakayama City, Japan; Department of Thoracic and Cardiovascular Surgery, Wakayama Medical University Hospital, Wakayama City, Japan; Department of Thoracic and Cardiovascular Surgery, Wakayama Medical University Hospital, Wakayama City, Japan

**Keywords:** Calcified thrombi, Aortic valve tumour, Tumour-like lesion, Atherosclerosis

## Abstract

Tumours or tumour-like lesions around the aortic valve are relatively rare and are difficult to diagnose. We report an interesting case of calcified thrombi in the Valsalva sinuses and coronary cusps that mimicked an aortic valve tumour. A 68-year-old man presented with a 20-mm calcified mass in the non-coronary and left-coronary cusps extending to their corresponding Valsalva sinuses, which was detected by echocardiography and contrast-enhanced computed tomography. The lesions were resected to establish the diagnosis and prevent systemic embolization. Intraoperative and histopathological examination revealed an atrophied non-coronary leaflet and calcified atherosclerotic lesions of the Valsalva sinuses and contiguous parts of the cusps, with ulceration and fibrin thrombi. The lesions were resected and aortic valve replacement was performed to avoid aortic valve dysfunction. The patient’s atrial fibrillation was controlled, and anticoagulants were discontinued 3 months postoperatively. Surgery to establish the diagnosis and to prevent systemic thromboembolism was thought to be reasonable, even in the absence of valvular dysfunction.

A 68-year-old man with paroxysmal atrial fibrillation and prostatic cancer was diagnosed with an aortic valve tumour by a primary physician, based on echocardiography. He had intermittent palpitations due to paroxysmal atrial fibrillation. Transthoracic echocardiography revealed a 20-mm isoechoic mass with an acoustic shadow around the non-coronary and left-coronary cusps, which had not been detected 8 years earlier. Laboratory findings did not show an elevation in tumour marker levels.

Contrast-enhanced computed tomography revealed a 20-mm, non-enhancing mass surrounded by calcification on the non-coronary and left-coronary cusps and their contiguous Valsalva sinuses (Fig. 1A and B). Transoesophageal echocardiography showed an immobile, isoechoic mass on the non-coronary and left-coronary cusps (Video 1), but there was no detection of aortic valve stenosis or regurgitation. Surgery was performed to establish the diagnosis of the tumour-like lesion and to avoid the risk of systemic embolization.

**Figure 1:**
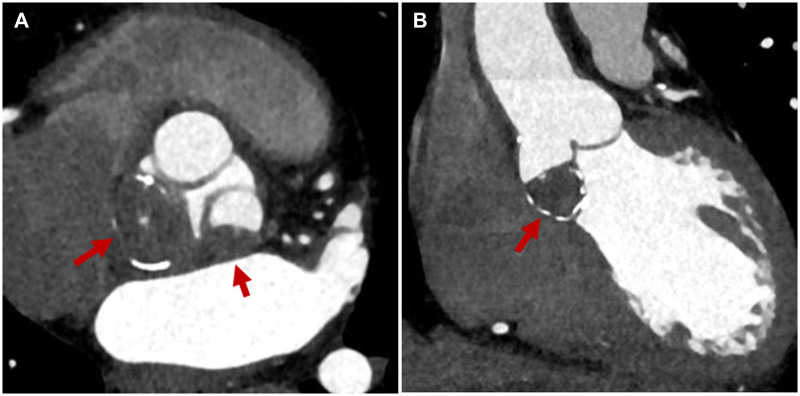
Contrast-enhanced computed tomography. (**A**) Axial view. (**B**) Coronal view. A non-enhancing mass surrounded by calcification is seen in the non-coronary cusp and Valsalva sinus and extending into the left-coronary cusp and Valsalva sinus. Red arrow: tumour-like lesion (A color version of this figure appears in the online version of this article).

The procedure was performed through a median sternotomy under cardiopulmonary bypass with ascending aortic and bicaval cannulation. When the standard aortic incision was made after antegrade cardioplegia, the non-coronary leaflet was noted to be slightly atrophied and shortened, and a part of the non-coronary cusp and the contiguous Valsalva sinus were distended with ulceration on the surface. Similar findings were noted in the left-coronary cusp and Valsalva sinus. The surfaces of the Valsalva sinuses were smooth and calcified (Fig. 2A and B). The distended coronary cusps and Valsalva sinuses were filled with dark red material (Fig. 2C). After extirpation of the contents, the defect was closed by sandwiching it with a felt strip. Considering the possibility of poor coaptation of the retracted aortic valve cusps after lesion resection, excision of the aortic valve leaflet and supra-annular implantation of a bioprosthetic valve (Inspiris RESILIA^®^ 21 mm, Edwards, US, Irvine, CA, USA) were performed. Pulmonary vein isolation was also performed for paroxysmal atrial fibrillation. On histopathological examination, the tumour-like lesion showed a fibrin thrombus (Fig. 2D), and the leaflet and a part of the thickened Valsalva wall surface showed myxoid degeneration. These findings were consistent with the diagnosis of calcified thrombi and atherosclerotic changes in the aortic valve.

**Figure 2:**
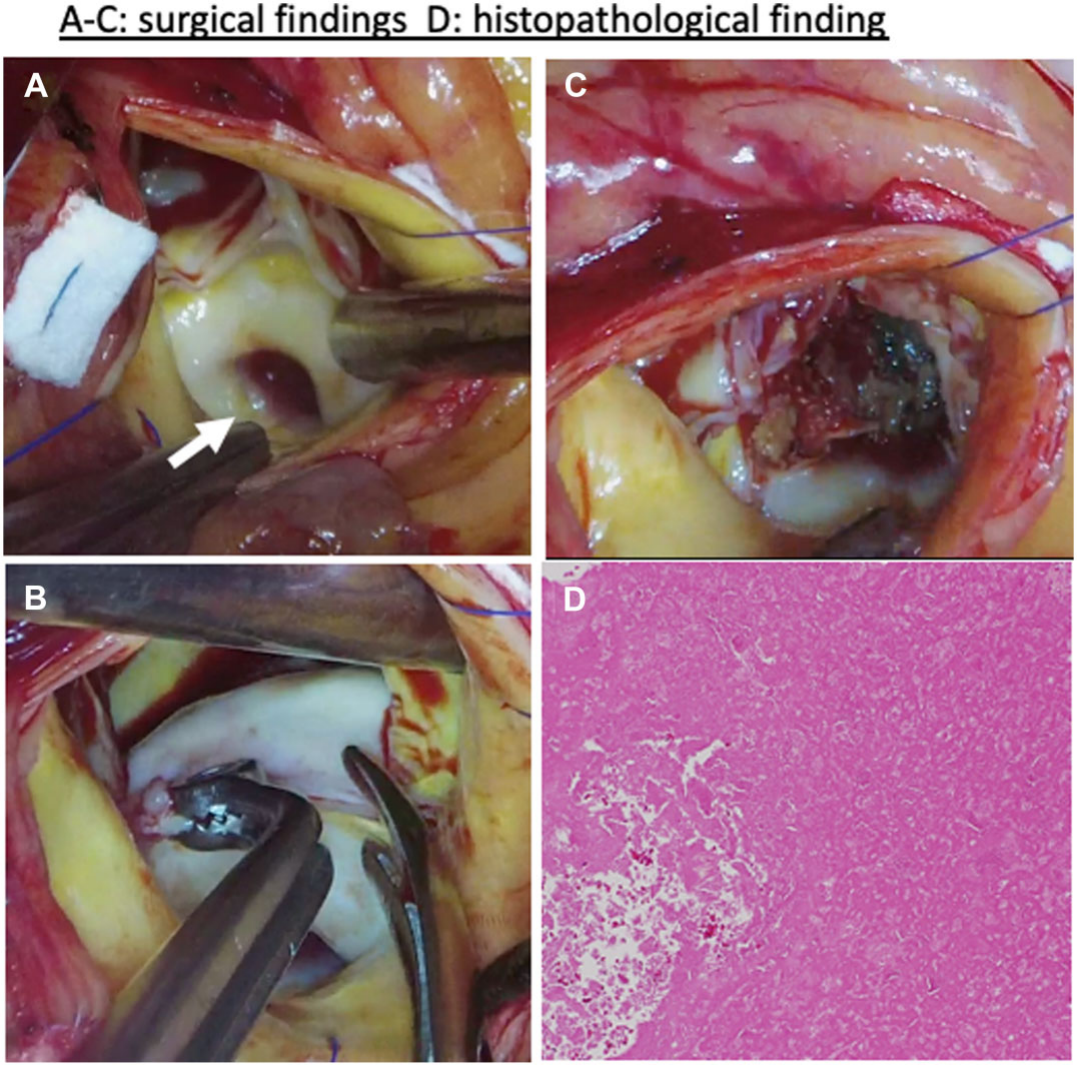
(**A–C**) Surgical findings. (**D**) Histopathological findings. (**A**) The non-coronary cusp and Valsalva sinus are distended and ulcerated on the smooth surface. (**B**) The distended Valsalva sinus and coronary cusp are hard due to calcification. (**C**) The distended lesions contain dark red thrombi. (**D**) These contents are mainly acidophilic, fibrin-like and amorphous, consistent with fibrin thrombi. White arrow: ulceration (A color version of this figure appears in the online version of this article).

The postoperative course was uneventful. Calcium channel and β-receptor blockade were used to achieve the blood pressure and heart rate of 120/80 mmHg and 60 bpm, respectively. Prothrombin time was controlled to maintain a target international normalized ratio of 2.0 with warfarin. The patient was discharged on the 10th postoperative day without any complications. Anticoagulants were discontinued 3 months postoperatively because there was no evidence of atrial fibrillation or aortic valve thrombus on transthoracic echocardiography.

There have been no previous reports of calcified thrombi and atherosclerotic changes in the coronary cusps and Valsalva sinuses, together forming a tumour-like lesion. They require differentiation from aortic valve tumours, such as calcified amorphous tumour, metastatic tumour or fibroelastoma because the treatment would be different [1–3]. Establishing the diagnosis is difficult, however, even by using various modalities. While most of the valvular thrombi described by Yuan *et al.* were mobile and without a stalk [4], the thrombi in our case did not have these characteristics.

Our case was of calcified tumour-like thrombi of the Valsalva sinuses and coronary cusps with a defect on the surface, which may have triggered a new thromboembolism. Making diagnosis and aggressive prevention of systemic and coronary embolization by surgery was thought to be appropriate, even without observation of aortic valve stenosis or insufficiency.
